# Do Drinking and Smoking Go Together?

**Published:** 1996

**Authors:** Saul Shiffman, Mark Balabanis

**Affiliations:** Saul Shiffman, Ph.D., is a professor of psychology and Mark Balabanis is a graduate student in clinical psychology at the Department of Psychology, University of Pittsburgh, Pittsburgh, Pennsylvania

**Keywords:** heavy AOD use, smoking, AODU (alcohol and other drug use) development, biochemical mechanisms, drug interaction, cessation of AODU, treatment, AOD abstinence, AODD (alcohol and other drug disorder) relapse

## Abstract

Heavy drinkers tend to be heavy smokers. Drinking appears to prompt smoking in real-life situations; whether smoking prompts drinking is uncertain. Alcohol-tobacco interactions are particularly important during attempts to achieve or maintain abstinence from either drug. Studies suggest that alcoholics who quit smoking are more likely to succeed in alcoholism treatment. However, data consistently demonstrate that alcohol consumption may precipitate smoking relapse.

Alcohol and tobacco seem to go together: Drinkers smoke and smokers drink. In addition, heavier drinkers tend to be heavier smokers. Use of alcohol and tobacco may be related in two ways. First, people who drink may also smoke (and vice versa); this is referred to as the between-person interaction. Second, people who use both drugs may use them together in the same situations. This is referred to as the situational interaction. Between-person and situational relationships are independent of each other: Each can theoretically occur without the other, and each can be explained by different potential mechanisms.

This article briefly reviews the nature of the relationships between drinking and smoking and examines their effect on alcoholism treatment and smoking cessation. Studies on smoking and drinking have been conducted with reference to three stages of drug use: (1) initiation (i.e., initial exposure to and use of a drug), (2) maintenance (i.e., ongoing use of a drug), and (3) cessation (i.e., addiction treatment or efforts to achieve and maintain abstinence). This article considers only the maintenance and cessation stages. Furthermore, it is not an exhaustive review of the literature but rather an illustration of some key issues.

## Between-Person Interaction

### The Maintenance Stage

Alcohol-tobacco interactions are prominent in the maintenance phase of addiction. Most smokers (i.e., 86 percent) drink alcohol, and smokers are 1.32 times more likely to drink than are non-smokers (Friedman et al. 1974). This relationship holds for both men and women but is slightly stronger among women. Conversely, smoking prevalence is 75 percent higher among drinkers than among nondrinkers (Abelson et al. 1973).

The link between drinking and smoking shows a dose-response relationship. That is, heavier drinkers tend to be heavier smokers (Friedman et al. 1991; Abrams et al. 1992). In addition, heavier drinkers also smoke each cigarette using more puffs and drawing in larger volumes of smoke (Keenan et al. 1990).

Existing evidence does not rule out the possibility that the overall correlation between drinking and smoking may reflect primarily the heavy end of the drinking-smoking continuum. For example, researchers have found smoking prevalence rates to be much higher among alcoholics than among nonalcoholics (Cyr and Wartmen 1988; DiFranza and Guerrera 1990) and even greater than 90 percent in some studies (Maletzky and Klotter 1974; Bobo 1992). Alcoholics may also smoke more heavily than nonalcoholic smokers. In one study, for example, the average smoking rate for alcoholics was 48.7 cigarettes per day (Maletzky and Klotter 1974), more than double the national average for smokers (U.S. Department of Health and Human Services 1988). Also, drinking and smoking rates correlated very highly among alcoholics but not among non-alcoholic control subjects (Maletzky and Klotter 1974). Conversely, alcoholism is much more prevalent among smokers than nonsmokers, particularly among women (DiFranza and Guerrera 1990; Covey et al. 1994).

Thus, prevalence as well as rates for drinking and smoking are linked in the general population, but the relationship appears particularly strong at higher levels of use: Heavy drinkers are likely to be heavy smokers and vice versa.

## Cessation and Relapse

The effect of heavy drinking on smoking cessation is uncertain. According to Carmelli and colleagues (1993), smokers who were lighter drinkers were more likely to report quitting smoking over a 16-year followup period. In addition, DiFranza and Guerrera (1990) reported that although alcoholics were as likely as nonalcoholic subjects to try to quit smoking, they were less likely to succeed. Similarly, smokers with past alcohol problems were less likely to quit smoking successfully (Hughes 1993). In a sample of alcoholics, Bobo and colleagues (1987) reported that severity of alcoholism was associated with failure to quit smoking. However, Hughes and Oliveto (1993) found that drinking rate had no influence on smoking relapse rates. Covey and colleagues (1993, 1994) similarly concluded that a history of alcoholism had no effect on smoking cessation; however, a subgroup of alcoholic men with histories of depression had significantly lower success rates. Thus, current or past alcoholism may impede smoking cessation efforts only in a subgroup of smokers.

Some addiction specialists have speculated that attempting or achieving smoking cessation might increase the risk of alcoholism treatment failure. Studies to date, conducted largely in real-life settings, suggest that alcoholics who quit smoking are *more* likely to succeed in alcoholism treatment (Shiffman and Balabanis 1995).

## Situational Interaction

### The Maintenance Stage

In laboratory studies, smokers increase smoking in response to alcohol consumption. In most early studies of this type, subjects consumed repeated doses of alcohol over 1 or more days. Smoking rate and amount of alcohol was assessed over whole days. This approach did not evaluate the effect of drinking on smoking at the moment of alcohol ingestion. However, subsequent studies demonstrated that single doses of alcohol increase smoking, either increasing the number of cigarettes smoked or by resulting in larger puffs when the number of cigarettes was held constant (Nil et al. 1984; Mintz et al. 1985).

Results of laboratory studies are supported by smokers’ self-reports that drinking prompts them to smoke in real-life situations. In one study, for example, 67 percent of smokers surveyed stated that they smoked when they drank (McKennell and Thomas 1967). However, studies have questioned the validity of self-reports as measures of smoking patterns (Shiffman 1993; Shiffman et al. 1994).

Shiffman and colleagues (1994) monitored the association between drinking and smoking using small palm-top computers with which subjects recorded the circumstances surrounding each smoking event and their mood at that time. The computers also “beeped” subjects at random to assess nonsmoking moments in similar fashion. Subjects were almost twice as likely to report recent drinking when they had been smoking. Moreover, alcohol consumption was the single strongest factor associated with smoking. Statistical analysis showed that the association between drinking and smoking could not be attributed to the effects of some third variable, such as mood.

### Does Smoking Prime Drinking?

No human studies have been conducted to assess whether smoking primes drinking (i.e., increases the probability of subsequent drinking). Only one animal study on this topic appears to exist. Potthoff and colleagues (1983) showed that rats implanted with continuous slow-release nicotine pellets doubled their daily alcohol intake. This result emphasizes the need for further research on this direction of influence.

### Cessation and Relapse

Data consistently demonstrate that alcohol consumption may precipitate smoking relapse. This has been demonstrated in several studies in which alcohol consumption increased the risk of smoking relapse, compared with situations in which the smoker was tempted but did not relapse (Shiffman 1982; Colletti et al. 1981; Cummings et al. 1985; Baer and Lichtenstein 1988). These results were confirmed in a recent study in which subjects’ relapse experiences were monitored using palm-top computers (Shiffman et al. in press). Drinking also was more common in situations in which people smoked their first cigarette after having temporarily abstained than at randomly sampled times. Some evidence indicates that the role of alcohol in promoting relapse increases later in abstinence (Cummings et al. 1985). No studies have addressed whether smoking acutely triggers alcohol relapse.

Researchers have speculated that alcohol may enhance relapse risk by releasing inhibitions that restrain smoking, thereby undermining coping (Shiffman 1982). However, the evidence for this is inconsistent (Curry and Marlatt 1985; Shiffman et al. in press), and other mechanisms also are plausible. For example, priming with one drug may reinstate the use of other drugs (de Wit and Stewart 1983; Stewart and Wise 1992). Conditioned associations between drinking and smoking also may underlie the association of drinking with smoking relapse (Abrams et al. 1992; Niaura et al. 1988).

## Mechanisms

Various explanations have been proposed to account for smoking-drinking associations. For example, use of alcohol and tobacco may reflect a common genetic propensity (Swan et al. 1994). Alcohol and tobacco also may induce cross-tolerance for each other (Burch et al. 1988). Tolerance is an aspect of addiction in which increasingly stronger doses of a drug are required to produce a given effect. Cross-tolerance is a phenomenon whereby tolerance to one drug induces tolerance to a different drug. Although these explanations may account for between-person differences, they do not account for situational effects (Swan et al. 1994).

Some researchers have proposed that people use tobacco when they drink to counteract alcohol’s depressant effects with nicotine’s stimulant effects (Lyon et al. 1975). In addition, alcohol may release inhibitions that restrain smoking (Shiffman 1982; Shiffman et al. 1994).^1^ These suggested mechanisms might explain situational aspects of the alcohol-tobacco association.

Finally, a stress-coping theory proposes that people resort to drug use—especially multiple drug use—when stressed beyond their capacity to cope (Wills and Shiffman 1985). High stress (a situational factor) or poor coping skills (a personal factor) may prompt a person to use alcohol and tobacco together to cope.

Differing theories of the alcohol-tobacco link need not be mutually exclusive. First, different theories may relate to different aspects of the same basic mechanism. For example, some of the association between drinking and smoking may result from common genetic factors in alcohol and tobacco use. However, these genetic contributions may be expressed as different aspects of personality that may themselves be heritable (Plomin 1990). Thus, genetic and personality explanations of the alcohol-tobacco link may not only be compatible—they may be two versions of the same theory. Second, different mechanisms may work simultaneously or even synergistically. For example, nicotine may exhibit both antianxiety and stimulant properties. Thus, people may smoke while drinking in an attempt to overcome stress more effectively while maintaining alertness and coordination (Lyon et al. 1975).

## Research Recommendations

Although much is known about the alcohol-tobacco link, much remains to be discovered. Future research should attempt to establish whether the observed relationship between alcohol and tobacco use holds only for heavy users or is truly dose-dependent throughout the range of use. In addition, research should determine the situational, as well as the individual, interactions of alcohol and tobacco. This approach requires studies in real-life environments as well as in the laboratory to ensure ecological credibility.

Research should focus on developing and testing theoretical models of the alcohol-tobacco link. Although researchers have speculated about mechanisms linking drinking and smoking, almost no studies have actually tested any theory.

Finally, the influence of tobacco on alcohol should be assessed, with particular attention given to the effect of smoking cessation on alcoholism treatment. Such research should clarify how smoking urges, feelings of deprivation relating to cigarettes, and symptoms of nicotine withdrawal may affect alcohol abstinence.

## Footnotes

1 In this context, the term “inhibitions” refers to the general concept of motivational and cognitive resolve that smokers use in order to refrain from smoking.
